# Tuberculosis screening of travelers to higher-incidence countries: A cost-effectiveness analysis

**DOI:** 10.1186/1471-2458-8-201

**Published:** 2008-06-05

**Authors:** Michael Tan, Dick Menzies, Kevin Schwartzman

**Affiliations:** 1 Respiratory Epidemiology Unit, Montreal Chest Institute, 3650 St. Urbain St., Montreal, Quebec, H2X 2P4, Canada; 2 Department of Epidemiology and Biostatistics, McGill University, Montreal, Quebec, Canada; 3 Respiratory Division, McGill University, Montreal, Quebec, Canada

## Abstract

**Background:**

Travelers to countries with high tuberculosis incidence can acquire infection during travel. We sought to compare four screening interventions for travelers from low-incidence countries, who visit countries with varying tuberculosis incidence.

**Methods:**

Decision analysis model: We considered hypothetical cohorts of 1,000 travelers, 21 years old, visiting Mexico, the Dominican Republic, or Haiti for three months. Travelers departed from and returned to the United States or Canada; they were born in the United States, Canada, or the destination countries. The time horizon was 20 years, with 3% annual discounting of future costs and outcomes. The analysis was conducted from the health care system perspective. Screening involved tuberculin skin testing (post-travel in three strategies, with baseline pre-travel tests in two), or chest radiography post-travel (one strategy). Returning travelers with tuberculin conversion (one strategy) or other evidence of latent tuberculosis (three strategies) were offered treatment. The main outcome was cost (in 2005 US dollars) per tuberculosis case prevented.

**Results:**

For all travelers, a single post-trip tuberculin test was most cost-effective. The associated cost estimate per case prevented ranged from $21,406 for Haitian-born travelers to Haiti, to $161,196 for US-born travelers to Mexico. In all sensitivity analyses, the single post-trip tuberculin test remained most cost-effective. For US-born travelers to Haiti, this strategy was associated with cost savings for trips over 22 months. Screening was more cost-effective with increasing trip duration and infection risk, and less so with poorer treatment adherence.

**Conclusion:**

A single post-trip tuberculin skin test was the most cost-effective strategy considered, for travelers from the United States or Canada. The analysis did not evaluate the use of interferon-gamma release assays, which would be most relevant for travelers who received BCG vaccination after infancy, as in many European countries. Screening decisions should reflect duration of travel, tuberculosis incidence, and commitment to treat latent infection.

## Background

In the United States, Canada, and other high-income countries, the proportion of tuberculosis (TB) cases among foreign-born individuals continues to increase [1,2]. The foreign-born have markedly higher TB incidence than persons born in low-incidence countries; the discrepancy is most evident in the first years after arrival, but persists for 20 years or longer [3]. This phenomenon highlights the impact of migration from higher- to low- incidence settings.

Travel to high-incidence settings is associated with increased incidence of TB disease and latent infection. Tuberculosis cases among established South Asian immigrants in London reflected return visits to their countries of origin [4]. Visits to parents' countries of origin were a consistent risk factor for latent TB infection among US-born children of immigrants [5,6]. Among 656 Dutch-born travelers to destinations with high TB incidence, the estimated incidence of latent TB infection was 4.2 per 100 person-years [7], a figure which equals or exceeds the annual risk of infection in the highest-incidence countries. Incidence was even higher for travelers engaged in health care work [7].

The acquisition of tuberculosis infection during travel is of particular concern, because of the high risk of active disease following new infection. The U.S. Centers for Disease Control and Prevention recommend that travelers "who anticipate possible prolonged exposure to tuberculosis" should undergo tuberculin tests before and after travel, while those "who anticipate repeated travel with possible prolonged exposure or an extended stay over a period of years in an endemic country" should undergo baseline two-step tuberculin testing, with subsequent annual tests if negative at baseline [8]. The Public Health Agency of Canada recommends pre-trip (two-step) and post-trip tuberculin tests for travelers who visit high-incidence countries for three months or longer, and for travelers engaged in any health care work in such countries [9].

To date, recommendations for traveler screening have been based on expert opinion. Using a decision analysis model, we conducted a cost-effectiveness analysis to evaluate four potential TB screening interventions for travelers to higher-incidence countries.

## Methods

### Screening strategies

We considered three screening strategies based on tuberculin skin testing, and one based on chest radiography (Table 1). Any positive tuberculin test was followed by clinical evaluation and chest radiography to identify or exclude active disease, with three mycobacterial cultures of spontaneous or induced sputum when indicated. Travelers diagnosed with active TB received standard drug treatment. Depending on the strategy, some or all travelers diagnosed with latent TB were prescribed a nine-month course of isoniazid (Table 1).

**Table 1:** Summary of screening strategies

Strategy	Pre-travel tuberculin skin test results		Interpretation of pre-travel results	Intervention
		
	Test 1	Test 2		
Pre-trip two step tuberculin test Treat converters	≥ 10 mm	N/A	Baseline latent tuberculosis infection (LTBI)	No intervention. Passive diagnosis of TB disease pursued if symptomatic post-travel.
	<10 mm	≥ 10 mm	Booster effect; no baseline LTBI	No intervention. Passive diagnosis of TB disease pursued if symptomatic post-travel.
	<10 mm	<10 mm	No LTBI	Post-trip tuberculin test. Converters prescribed isoniazid.
Pre-trip two step tuberculin test Treat reactors and converters	≥ 10 mm	N/A	Baseline LTBI	Prescribe isoniazid.
	<10 mm	≥ 10 mm	Booster effect	No intervention. Passive diagnosis of TB disease pursued if symptomatic post-travel
	<10 mm	<10 mm	No LTBI	Post-trip tuberculin test. Converters prescribed isoniazid
Post-trip tuberculin test	None		None	One post-trip tuberculin skin test. All reactors (≥ 10 mm) prescribed isoniazid.
Post-trip chest x-ray	None		None	Chest x-ray post-trip. Persons with radiographic abnormalities compatible with TB, negative workup for active TB, and TST ≥ 5 mm prescribed isoniazid.
No screening	No intervention before or after trip. Passive diagnosis of TB disease pursued if symptomatic post-travel.			

For comparison, we included a strategy of no screening–returning travelers presented to health facilities if they developed TB symptoms.

### The decision analysis model

**Figure 1:**
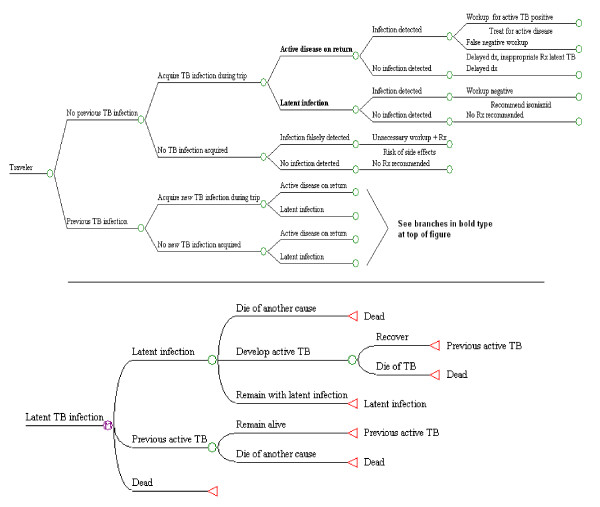
**Schematic view of decision trees**. The top portion of the figure summarizes the sequence of events leading to possible tuberculosis infection after travel, and the use of post-travel screening (by skin testing or chest radiography) to detect latent infection or active disease. If two-step tuberculin skin testing is undertaken before travel, then post-travel testing is limited to travelers with negative baseline results, while those with positive baseline results may be referred for isoniazid treatment, as described in the text and in Table 1. Persons who return from travel with latent infection enter the Markov process illustrated in the lower portion of the figure, although those who receive isoniazid may be cured of latent infection and face no future risk of reactivation.

We developed a decision analysis model, using DATA version 4.0 for Health Care (TreeAge, Williamstown, MA). The model incorporated multiple Markov processes, including the following health states: uninfected, latent TB infection, active TB disease, previous active disease (recovered), and dead. Transitions reflected risks of acquiring infection; risks of progression to active disease depending on time since infection, and on treatment for latent infection; cure rates with treatment of latent and active TB; and risk of mortality from TB or other causes. Figure 1 provides an overview. Further details are available elsewhere [10].

We simulated tuberculosis-related events over 20 years, beginning at departure, among 1000 travelers from the United States or Canada who visited Mexico, the Dominican Republic, or Haiti. We considered separately 1) travelers born in the United States or Canada; and 2) travelers residing in the United States or Canada, but born in Mexico, the Dominican Republic, or Haiti, who made return visits to their countries of birth. We considered these countries because they are frequent travel destinations, and frequent countries of origin for foreign-born persons in the United States and Canada. They span a range of TB incidence relevant to many potential destinations.

Main outcomes were active tuberculosis cases, and costs. While deaths were initially modeled, TB-related deaths were so rare that differences between strategies were virtually non-existent, so deaths were not further analyzed. Expected TB cases and costs were discounted at an annual rate of 3% [11]. The analysis was conducted from the health care system perspective. Model assumptions are summarized in Table 2.

**Table 2:** Model assumptions

	**Base value**	**Range**	**Sources**
Annual risk of progression from latent tuberculosis (LTBI) to TB disease			
Among persons newly infected during travel			
First two years after infection	0.025	0.02 – 0.05	[33]
Subsequently	0.001	0.001 – 0.002	[34]
Among persons with LTBI before travel, given			
No underlying radiographic abnormalities [see below]	0.001		[34]
Underlying radiographic abnormalities [see below]	0.0066		[34]
Reduction in risk afforded by:			
Full 9 months of isoniazid, given drug-sensitive latent infection	90%		[35]
<6 months isoniazid	0%		[36]
Previous LTBI, among persons who are reinfected during travel	79%		[37]

Prevalence of isoniazid resistance			
Mexico	0.072	0.072 – 0.12	[38, 39]
Dominican Republic	0.198	--	[40]
Haiti	0.159	--	[41]

Active TB			
Proportion of travelers with active TB symptomatic upon return	0.27	0.11 – 0.44	[21, 26]
Probability of hospitalization given active TB diagnosed after symptoms	0.8		[42]
Probability of hospitalization given active TB diagnosed through screening	0.5		[21, 22]
Probability of completing full anti-TB therapy	1.0		assumed
Risk of major side effect with full anti-TB therapy	0.051	0.01 – 0.1	[43]
Probability of death, given major treatment side effect	0.015	0.001 – 0.032	[43–45]

Treatment of latent TB infection			
Probability of completing 9 months isoniazid	0.647	0.62 – 1	[10, 46]
Probability of major side effect with isoniazid	0.003		[43–45]
Probability of death, given major treatment side effect	0.015	0.001 – 0.032	[43–45]

Tuberculin skin testing			
Probability of boosting, given previous BCG vaccination	0.25		[17]
Specificity for LTBI	0.875	-	[17]
Sensitivity for LTBI	0.99	-	[47, 48]
Sensitivity for active TB	0.88	-	[48]
Probability of loss to follow-up between pre- and post- travel evaluations, for repeat testing strategies	0.34		[7]

Probability of abnormal chest X-ray upon return from travel			
With preexisting LTBI	0.11	0.07 – 0.15	[33, 34, 49, 50]
With LTBI newly acquired during travel	0		assumed
With active TB	0.95	0.9 – 1	[51, 52]

Sputum cultures (3) for *M. Tuberculosis*			
Specificity	0.99		[51]
Sensitivity	0.9		[53]

Costs for TB screening and care in the US (expressed in 2005 US dollars)			
Initial clinic visit	$68		[54, 25]
Tuberculin skin test	$12		[42, 23]
Follow-up clinic visit after tuberculin test	$36		[42]
Chest radiograph with reading	$36		[42]
Isoniazid, 9 months supply	$25		[54]
7 outpatient clinic visits during isoniazid treatment	$385		[54]
Major adverse reaction to isoniazid	$9,834		[54]
3 sputa for AFB smear and culture, after abnormal CXR	$126		[55, 56]
Inpatient treatment of active TB disease	$9,061		[57]
Outpatient treatment of active TB disease	$2,600		[42]
Contact investigation and management, per active TB case (identified via screening)	$4,483		[42, 24]

### Travelers

In the base case analysis, we assumed that travelers were 21 years old and HIV-seronegative, and that those born abroad had moved to the United States or Canada at age 11. Trips lasted three months. During travel, visitors faced the same risks of TB infection as the local population, with the same probability of infection with a drug-resistant strain–as highlighted in Table 2[7]. The estimated annual risks of TB infection in Mexico, the Dominican Republic, and Haiti were 0.3%, 0.8%, and 2.6% respectively, using the reported incidence of smear-positive disease in those countries in 2004 [12] and the Styblo formula [13].

For travelers born abroad, the likelihood of latent TB infection before travel reflected the annual risk in the country of origin and the age (11 years) at which they were assumed to have emigrated. Background mortality rates were based on US and Canadian life tables [14,15]. All travelers were assumed to be HIV-seronegative, reflecting the low prevalence of HIV infection in the US and Canadian general population, and screening practices for HIV among prospective immigrants to the US and Canada. Clinical considerations would be very different for HIV-infected travelers, who should undergo baseline TB screening at the time of HIV diagnosis.

### BCG vaccination

We considered that travelers born in Canada or the US had not received the Bacille Calmette-Guérin (BCG) vaccine. Travelers born in Mexico, the Dominican Republic, or Haiti were assumed to have received BCG vaccination during infancy only, with probabilities equal to reported coverage rates in those countries for the year 1984 (47%, 43%, and 71% respectively) [16]. BCG vaccination during infancy did not provide any protection with respect to active TB after travel, and we assumed it did not lead to false-positive first-step tests [17,18]. However, we assumed a 25% probability of boosting on the second-step test among persons who had been BCG vaccinated [17,18].

### Secondary transmission

In addition to active TB cases among travelers themselves, the analysis also incorporated latent infection and active disease among their contacts. Our estimates reflected the mathematical model of TB transmission developed by Salpeter and Salpeter [19], the relative contagiousness of smear-positive and smear-negative source patients with pulmonary disease [20], and the proportion of smear-positive disease among persons diagnosed passively vs. actively [21]. On these grounds, we estimated that persons with active TB diagnosed passively on the basis of symptoms infected 3.5 contacts each, with 0.7 cases of secondary active disease, while persons with active TB diagnosed by screening infected 1.5 contacts each, with 0.3 cases of secondary active disease [22].

### Loss to follow-up

Based on the Dutch study of travelers to high-incidence countries, we assumed that 34% of travelers would be lost to follow-up post-trip [7].

### Costs

Component costs for US travelers were based on two US surveys of TB-related health care costs [23,24]. Additional US costs were obtained from the fee schedule published by the US Centers for Medicare and Medicaid Services [25]. Component costs for Canadian travelers were derived from earlier program evaluations and cost-effectiveness analyses from Montreal [22,26]. We assumed that 80% of persons with active TB diagnosed because of symptoms would be hospitalized, while the corresponding figure would be 50% for those diagnosed by screening [21,26].

All costs were expressed in year 2005 US dollars (for US travelers) or year 2005 Canadian dollars (for Canadians) [27,28], $1 Canadian (2005) = $0.89 US (2005). Table 2 lists US cost estimates.

### Sensitivity analyses

We ran one-way sensitivity analyses for all assumed parameters. We repeated the analysis using alternative visit durations (6–24 months) and traveler ages, and with exclusion of cases and costs related to secondary transmission. Two-way sensitivity analysis evaluated the simultaneous influence of visit duration and annual risk of infection in the destination country.

### Research ethics

As this study did not involve human subjects, approval from the institutional ethics review board was not required.

## Results

### Base case analysis

For every 1,000 US-born travelers who visited Mexico for three months, the expected number of TB cases was 1.0 and the expected cost $7,428 over the 20-year simulation, in the absence of any intervention (Table 3). Of the interventions considered, the single post-trip tuberculin skin test was both the cheapest ($47,082 per 1,000 travelers) and the most effective (0.2 cases prevented per 1,000 travelers) in this traveler group, but this intervention cost an estimated $161,196 per case prevented, relative to no intervention. For US-born travelers to the Dominican Republic or Haiti, the same strategy was again cheapest and most effective, but with an estimated cost of $102,745 or $36,931 per case prevented, respectively. For travelers born in the destination countries, the single post-trip tuberculin test was also the preferred strategy (Table 3).

**Table 3:** Base case analysis: Cost-effectiveness of screening 21 year-old travelers for 3-month trips

Traveler's destination Traveler group: Annual risk of infection Screening strategy from least to most expensive	Expected costs per 1,000 (2005 USD)	Expected cases per 1,000	Incremental cost per 1,000^†^	Incremental cases prevented per 1,000^†^	Incremental cost per case prevented^†^
**Travelers to Mexico**					
**US-born**: Annual infection risk 0.3%					
No screening	$7,428	1.0	-	-	-
Post-trip TST	$47,082	0.8	$39,654	0.2	$161,196
Post-trip chest x-ray	$58,972	0.9	$11,890	(0.1)	Dominated
Pre & post trip TST; treatment for reactors	$68,644	0.8	$9,672	0.1	Dominated
Pre & post trip TST; treatment for converters only	$68,741	0.9	$97	(0.1)	Dominated
**Mexican-born**: Annual infection risk 0.3%					
No screening	$9,855	1.3	-	-	-
Post-trip TST	$57,666	1.0	$47,811	0.3	$143,578
Post-trip chest x-ray	$61,087	1.1	$3,421	(0.1)	Dominated
Pre & post trip TST; treatment for converters only	$71,335	1.2	$10,248	(0.1)	Dominated
Pre & post trip TST; treatment for reactors	$74,372	1.1	$3,037	0.1	Dominated

**Travelers to Dominican Republic**					
**US-born**: Annual infection risk 0.8%					
No screening	$13,226	1.7	-	-	-
Post-trip TST	$51,961	1.3	$38,735	0.4	$102,745
Post-trip chest x-ray	$64,678	1.6	$12,718	(0.3)	Dominated
Pre & post trip TST; treatment for reactors	$73,792	1.4	$9,114	0.2	Dominated
Pre & post trip TST; treatment for converters only	$73,889	1.5	$97	(0.1)	Dominated
**Dominican-born**: Annual infection risk 0.8%					
No screening	$25,423	3.3	-	-	-
Post-trip TST	$73,523	2.6	$48,100	0.7	$65,264
Post-trip chest x-ray	$75,711	2.9	$2,188	(0.3)	Dominated
Pre & post trip TST; treatment for reactors	$86,604	2.8	$10,893	0.1	Dominated
Pre & post trip TST; treatment for converters only	$89,568	3.2	$2,964	(0.4)	Dominated

**Travelers to Haiti**					
**US-born**: Annual infection risk 2.6%					
No screening	$34,041	4.4	-	-	-
Post-trip TST	$68,756	3.5	$34,715	0.9	$36,931
Post-trip chest x-ray	$85,163	4.3	$16,407	(0.8)	Dominated
Pre & post trip TST; treatment for reactors	$91,797	3.8	$6,634	0.5	Dominated
Pre & post trip TST; treatment for converters only	$91,893	3.9	$96	(0.1)	Dominated
**Haitian-born: **Annual infection risk 2.6%					
No screening	$73,594	9.6	-	-	-
Post-trip chest x-ray	$119,983	8.5	$46,389	1.1	$40,585*
Post-trip TST	$121,073	7.4	$1,090	1.1	$1,014
Pre & post trip TST; treatment for reactors	$133,556	8.1	$12,484	(0.7)	Dominated
Pre & post trip TST; treatment for converters only	$135,395	9.2	$1,839	(1.1)	Dominated

^†^For each type of traveler, strategies are listed by increasing cost. In the three rightmost columns, the calculation of incremental values (e.g., incremental cost per 1,000) is based on the comparison between each strategy and its next least costly alternative (e.g., a row might show the difference in cost between the second least costly strategy and the least costly strategy; the next row below would then show the cost difference between the third and second least costly strategies; and so on). Values in parentheses indicate an increase in expected cases, instead of cases prevented. "Dominated" means that another strategy is available that is both less costly and effective in preventing more TB cases.

*Dominated by extended dominance. While post-trip chest X-ray is the least costly option, another choice is available which is more attractive on an incremental cost per case prevented basis. The incremental cost per TB case prevented of post-trip TST, compared to no screening, is $21,406.

Costs were different for travelers departing from Canada, but outcomes were similar. Hence as for US travelers, the single post-trip tuberculin test prevented the most active TB cases. This strategy was also cheaper than the other interventions. However, the incremental cost per case prevented was consistently higher than for US travelers–primarily reflecting lower costs for TB-related hospitalization in the Canada. For instance, among Canadian-born travelers visiting Haiti for 3 months, it was $44,389 Canadian ($39,506 US) per TB case prevented.

### Sensitivity Analyses

Regardless of trip duration, the single post-trip tuberculin test was preferred, as it was consistently the cheapest intervention per case prevented. All interventions became more cost-effective with increasing length of stay abroad. For example, among US-born travelers to Haiti, with a 24-month trip, screening became cost saving (Table 4). In fact, the single post-trip tuberculin testing strategy was predicted to result in cost savings for US-born travelers staying in Haiti for 21.8 months or longer. For Haitian-born travelers from the US returning to Haiti, and for travelers to Mexico or the Dominican Republic, this strategy was not expected to produce cost savings for stays up to 24 months.

**Table 4:** Sensitivity analysis: Incremental cost per tuberculosis case prevented by post-trip tuberculin testing (versus no screening)

		**All costs in 2005 US dollars**					
		
		**Mexico**		**Dominican Republic**		**Haiti**	
		Annual risk of infection 0.3%		Annual risk of infection 0.8%		Annual risk of infection 2.6%	
			
		US-born	Mexican-born	US-born	Dominican-born	US-born	Haitian-born
Base case		$161,196	$143,578	$102,745	$65,264	$36,931	$21,406
Increase traveler age from 21 years†	to 35 years	$163,779	$148,268	$103,993	$67,644	$37,482	$22,651
	to 55 years	$178,237	$164,591	$113,168	$76,263	$40,989	$26,537
Increase trip duration from 3 months†	to 6 months	$111,832	$60,889	$16,787	$108,476	$48,731	$15,261
	to 12 months	$67,592	$71,720	$31,427	$31,217	$5,426	$8,554
	to 24 months	$35,872	$41,050	$13,622	$16,510	Cost saving	$2,740
Change isoniazid completion rate from 65%†	to 50%	$208,386	$185,056	$132,752	$84,964	$48,813	$29,113
	to 100%	$103,191	$93,192	$65,368	$41,179	$22,017	$12,007

†All other parameters held constant at base case value

For Canadian-born travelers to Haiti, the post-trip tuberculin test became cost saving for trips over 17.4 months. For all other travelers departing from Canada, no cost savings were predicted for trips up to 24 months.

**Figure 2:**
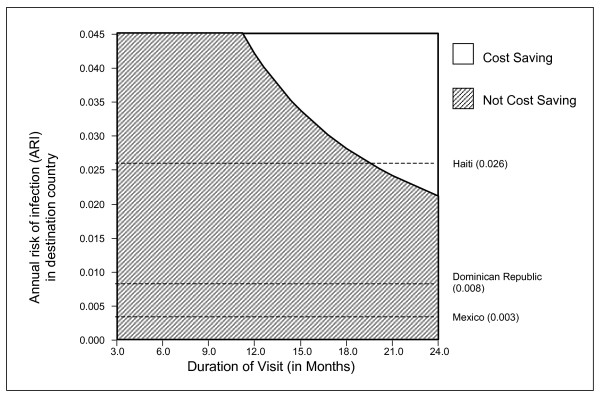
**Two-way sensitivity analysis examining the impact of travel duration and annual risk of infection on potential cost savings with the post-trip tuberculin testing strategy, for US-born travelers**. During longer trips and/or high annual infection risks, this approach becomes cost-saving, relative to no screening. The two-way sensitivity analysis assumes a 7.2% prevalence of isoniazid resistance, as in Mexico. Hence the threshold trip duration for cost savings among travelers facing infection risks as in the Dominican Republic and Haiti is shorter than in the base case scenario. The base case scenario used the higher prevalence of isoniazid resistance which has been documented in those countries (19.8% and 15.9%).

As the risk of infection increased, all interventions became more cost-effective; the single post-trip tuberculin test remained the preferred strategy. The results of a formal two-way sensitivity analysis evaluating the impact of infection risk and visit duration on the post-trip screening strategy are shown in Figure 2. The post-trip TST strategy became cost-saving for longer visit durations, very high infection risks, or both.

With older traveler age, the cost per case prevented increased. This reflected the increased background mortality rate, and the higher risk of side effects with treatment of latent infection. However, this effect was relatively modest (Table 4).

We repeated the analysis with the same base case parameters, except that secondary transmission was ignored. This led to fewer expected cases (particularly in the absence of any intervention) but screening costs were unchanged. Hence the costs of all interventions increased relative to the number of cases prevented. For example, the cost per case prevented among US-born travelers visiting Haiti for 3 months increased from $36,931 to $41,598.

Any refusal to undergo screening would reduce the public health impact of all interventions. Failure to return for follow-up after skin testing would reduce the cost-effectiveness of each intervention. This was particularly true for the two strategies which involved pre-travel tuberculin tests, so that these strategies were even less attractive when we increased the probability of loss to follow-up above the base case value of 34%.

Regardless of the assumed completion rate for isoniazid treatment, the single post-trip tuberculin test remained the preferred intervention. However, the completion rate substantially affected the number of cases prevented by each intervention, and thus the cost per case prevented (Table 4).

## Discussion

For three-month trips to most destinations, tuberculosis screening by any method is expensive, and yields a limited absolute reduction in subsequent TB incidence. For trips of six or more months to high-incidence destinations, a single post-trip tuberculin skin test is reasonably cost-effective, and may be cost-saving under some conditions. For such trips, screening is particularly relevant for health care workers, and for travelers visiting friends and relatives. On the other hand, screening would be even less cost-effective for typical trips of 1–2 weeks, and for visits to tourist resorts.

The single post-trip tuberculin test is clearly preferable to repeated testing pre and post-travel, and to chest radiography, across a wide variety of settings and assumptions. The single test is more effective, in that there may be fewer opportunities for losses to follow-up than with the repeated pre-travel screens (an important assumption), and many more cases of new infection diagnosed than with chest radiography.

A post-travel tuberculin test alone cannot distinguish remote from recently acquired infection. However, for travelers born in the United States or Canada who visit high-incidence countries, positive post-travel tests will often reflect infection acquired during travel. For travelers born in high-incidence countries, positive post-travel tests may reflect longstanding infection, but treatment will still be beneficial–particularly for younger travelers–and persons with newly acquired infection will still be referred for treatment. We used a 10 mm threshold, as would ordinarily be used for HIV-seronegative persons moving to the United States or Canada from abroad. We did not consider the 5 mm cutoff used for contact investigation, for persons with HIV infection, or those with other major risk factors for reactivation, as such individuals represent a small minority of travelers and were not the focus of this analysis.

The decision analysis allowed us to explicitly model the different elements of each intervention, and key parameters influencing costs and clinical impact. Predicted costs and numbers of tuberculosis cases varied considerably under different scenarios, notably infection risk and travel duration. However, the single post-trip tuberculin test remained the preferred intervention across all scenarios considered.

We limited the analysis to the perspective of the health care system, since we did not have reliable estimates for lost earnings and out-of-pocket costs associated with screening. Adoption of this perspective meant that lost earnings and patient costs related to active TB were ignored, but it also meant that we did not consider time lost from work to undergo screening. Given the small number of active cases prevented, relative to the number of travelers screened, it is possible that incorporation of time lost from work would actually increase costs per case prevented. This is particularly likely for the two strategies which involved two-step skin tests before departure.

Another limitation of this analysis was the use of tuberculosis cases as the primary outcome measure. The ideal is to express effectiveness in terms of quality-adjusted survival, but TB-related mortality is extremely rare among HIV-negative persons treated in the United States and Canada, while reliable data reporting quality adjustment for TB-related morbidity are scarce [29].

Our analysis was limited to travelers leaving from and returning to the United States or Canada, and used cost estimates from those countries. The expected costs of the four screening strategies for travelers from other low-incidence countries would vary according to the costs of the individual screening components, of TB drugs, and of TB care in those countries. However, if the relative costs of screening versus TB care are similar to those in the US and Canada, then the single post-trip test would likely remain the preferred option.

For three month visits to Mexico and the Dominican Republic, the estimated cost per case prevented by the post-trip tuberculin testing strategy compared poorly to established interventions, such as chest radiographic screening of immigrant applicants entering Canada [26] where the estimated cost was $48,473 Canadian ($43,141 US). For three-month visits to Haiti, the cost per case prevented was more comparable, though still much greater than the estimated $561 US per case prevented through screening high-risk kindergarten pupils in the US [24].

A potential limitation of this analysis was the estimate of annual risk of infection, obtained by applying the Styblo formula to reported incidence of smear-positive disease in the three countries we considered. This formula was derived before the advent of the HIV epidemic and may not accurately estimate the precise infection risk in each country. However, we considered a wide range of infection risk, with consistent results in terms of the preferred strategy.

In clinical practice, travelers might be more often lost to follow-up than the 34% lost to follow-up in the Dutch cohort study [7]. In that setting, travelers volunteered to participate in a research study, underwent two-step testing before departure, and were actively followed up after their return. Loss to follow-up after travel substantially diminishes the cost-effectiveness of strategies with baseline testing pre-travel, since anyone lost to follow-up incurs screening costs without benefit. With the single tuberculin test, travelers who elect not to be tested after their return neither incur screening costs nor derive benefit. Hence the absolute public health impact is reduced, but not the cost-effectiveness.

One alternative intervention that would reduce the inconvenience of repeated testing and reading is the use of interferon-gamma release assays. These also potentially offer enhanced specificity for the diagnosis of latent TB in individuals who received BCG vaccination after infancy [30,31]. Because of the unit costs of these tests, their use could be considerably more expensive than that of tuberculin testing for most travelers. However, they could reduce the frequency of unnecessary treatment among travelers who were BCG-vaccinated after infancy. In this analysis we focused on individuals born in the United States or Canada–who are not ordinarily vaccinated–and on foreign-born persons vaccinated only at birth.

Use of an interferon-gamma release assay would be most relevant, and potentially cost-effective, for travelers born in countries where the BCG vaccination is administered after infancy, as in many European countries. A single post-trip test with one of the newer-generation interferon-gamma release assays might be considered as an alternative in such travelers, to avoid potential confounding by BCG vaccination after infancy. However, a formal cost-effectiveness evaluation of this option was beyond the scope of the current analysis. Interested readers are referred to a recently published cost-effectiveness analysis which compared interferon-gamma release assays with tuberculin tests and chest X-rays for TB screening, among close contacts and immigrants. The analysis highlighted the potential utility of interferon-gamma release assays as confirmatory tests for latent infection, after positive tuberculin tests in persons from countries where BCG is repeatedly administered [32].

The decision to screen for latent TB among travelers must take into account the duration of the trip, the risk of infection in the destination country, and the likelihood of exposure (e.g. health care workers). For healthy travelers visiting destinations with high TB incidence, it may be reasonable to consider screening for trips lasting 6 or more months. For individual travelers, the decision must also account for factors that increase the risk of subsequent active TB (e.g. immune suppression, diabetes) as well as factors that increase the risk of treatment toxicity. If screening is undertaken, travelers should plan a single tuberculin test after returning home.

## Competing interests

The authors declare that they have no competing interests.

## Authors' contributions

MT aided in conception and design of the study, developed the decision trees and conducted the analyses reported in this manuscript, and wrote the first draft of the manuscript. DM aided in conception and design of the study, aided in supervision of the decision analyses, and reviewed and rewrote the manuscript critically for important intellectual content. KS aided in conception and design of the study, supervised the development of the decision trees and the ensuing analyses, and reviewed and rewrote the manuscript critically, for important intellectual content. All authors have read and approved the final manuscript.

## Pre-publication history

The pre-publication history for this paper can be accessed here:



## References

[B1] Centers for Disease Control and Prevention (2005). Reported Tuberculosis in the United States, 2004. Centers for Disease Control and Prevention, Division of Tuberculosis Elimination (DTBE).

[B2] Ellis E, Gallant V, Scholten Dx, Miron M, Public Health Agency of Canada (2006). Tuberculosis in Canada 2004 Pre-release. Public Health Agency of Canada.

[B3] Medical Research Council tuberculosis and Chest Diseases Unit (1980). National survey of tuberculosis notifications in England and Wales 1978–9. Br Med J.

[B4] McCarthy OR (1984). Asian immigrant tuberculosis–the effect of visiting Asia. Br J Dis Chest.

[B5] Lobato MN, Hopewell PC (1998). Mycobacterium tuberculosis infection after travel to or contact with visitors from countries with a high prevalence of tuberculosis. Am J Respir Crit Care Med.

[B6] Saiman L, San GP, Schulte J, Vargas MP, Kenyon T, Onorato I (2001). Risk factors for latent tuberculosis infection among children in New York City. Pediatrics.

[B7] Cobelens FG, Van DH, Draayer-Jansen IW, Schepp-Beelen AC, van Gerven PJ, van Kessel RP (2000). Risk of infection with Mycobacterium tuberculosis in travellers to areas of high tuberculosis endemicity. Lancet.

[B8] Centers for Disease Control and Prevention Health Information for International Travel – The "Yellow Book": Chapter 4 – Prevention of Specific Infectious Diseases: Tuberculosis (2005–2006). CDC Travelers' Health: Yellow Book.

[B9] Public Health Agency of Canada, Committee to Advise on Tropical Medicine and Travel (1997). The risk and prevention of tuberculosis in travelers. Public Health Agency of Canada, Canada Communicable Disease Report.

[B10] Schwartzman K, Oxlade O, Barr RG, Grimard F, Acosta I, Baez J (2005). Domestic returns from investment in the control of tuberculosis in other countries. N Engl J Med.

[B11] Gold MR, Siegel JE, Russell LB, Weinstein MC, eds (1996). Cost-effectiveness in health and medicine.

[B12] WHO Global tuberculosis control surveillance, planning, financing WHO Report 2006. WHO, Programmes and Projects, Tuberculosis.

[B13] Styblo K (1985). The relationship between the risk of tuberculous infection and the risk of developing infectious tuberculosis. Bulletin of the International Union against Tuberculosis.

[B14] Centers for Disease Control and Prevention, National Center for Health Statistics United States Life Tables 2002. CDC National Center for Health Statistics: National Vital Statistics Reports.

[B15] Statistics Canada Duchesne D. Life tables, Canada, provinces and territories, 1995–1997. Statistics Canada, Summary Tables.

[B16] World Health Organization WHO-UNICEF estimates of BCG coverage. WHO, Immunization, surveillance, assessment & monitoring.

[B17] Menzies RI, Vissandjee B (1992). Effect of Bacille Calmette-Guerin vaccination on tuberculin reactivity. Am Rev Respir Dis.

[B18] Farhat M, Greenaway C, Pai M, Menzies D (2006). False positive tuberculin tests: what is the absolute effect of BCG and non-tuberculous mycobacteria. Int J Tuberc Lung Dis.

[B19] Salpeter EE, Salpeter SR (1998). Mathematical model for the epidemiology of tuberculosis, with estimates of the reproductive number and infection-delay function. Am J Epidemiol.

[B20] Behr MA, Warren SA, Salamon H, Hopewell PC, Ponce de LA, Daley CL (1999). Transmission of Mycobacterium tuberculosis from patients smear-negative for acid-fast bacilli. Lancet.

[B21] Mubareka S, Perrault M, Rocher I, Menzies D, Schwartzman K (2004). Tuberculosis diagnosed by active vs. passive case-finding. Am J Respir Crit Care Med.

[B22] Schwartzman K, Menzies D (2000). Tuberculosis screening of immigrants to low-prevalence countries. A cost-effectiveness analysis. Am J Respir Crit Care Med.

[B23] Schechter CB, Rose DN, Fahs MC, Silver AL (1990). Tuberculin screening: cost-effectiveness analysis of various testing schedules. Am J Prev Med.

[B24] Mohle-Boetani JC, Miller B, Halpern M, Trivedi A, Lessler J, Solomon SL (1995). School-based screening for tuberculous infection. A cost-benefit analysis. JAMA.

[B25] US Department of Health and Human Services Medicare Payment Systems and Coding Files. HHS, Centers for Medicare & Medicaid Services.

[B26] Dasgupta K, Schwartzman K, Marchand R, Tennenbaum TN, Brassard P, Menzies D (2000). Comparison of cost-effectiveness of tuberculosis screening of close contacts and foreign-born populations. Am J Respir Crit Care Med.

[B27] U S Bureau of Labor Statistics Consumer Price Index Inflation Calculator. http://data.bls.gov/cgi-bin/cpicalc.pl.

[B28] Bank of Canada, Rates and Statistics Inflation Calculator. http://www.bankofcanada.ca/en/rates/inflation_calc.html.

[B29] Dion MJ, Tousignant P, Bourbeau J, Menzies D, Schwartzman K (2004). Feasibility and reliability of health-related quality of life measurements among tuberculosis patients. Qual Life Res.

[B30] Ferrara G, Losi M, D'Amico R, Roversi P, Piro R, Meacci M (2006). Use in routine clinical practice of two commercial blood tests for diagnosis of infection with Mycobacterium tuberculosis: a prospective study. Lancet.

[B31] Pai M, Riley LW, Colford JM (2004). Interferon-gamma assays in the immunodiagnosis of tuberculosis: a systematic review. Lancet Infect Dis.

[B32] Oxlade O, Schwartzman K, Menzies D (2007). Interferon-gamma release assays and TB screening in high-income countries: a cost-effectiveness analysis. Int J Tuberc Lung Dis.

[B33] Ferebee SH (1969). Controlled chemoprophylaxis trials in tuberculosis. Adv Tuberc Res.

[B34] Nolan CM, Elarth AM (1988). Tuberculosis in a cohort of Southeast Asian Refugees. A five-year surveillance study. Am Rev Respir Dis.

[B35] Comstock GW (1999). How much isoniazid is needed for prevention of tuberculosis among immunocompetent adults?. Int J Tuberc Lung Dis.

[B36] International Union Against Tuberculosis Committee on Prophylaxis (1982). Efficacy of various durations of isoniazid preventive therapy for tuberculosis: five years of follow-up in the IUAT trial. Bull World Health Organ.

[B37] Menzies D (1997). Issues in the management of contacts of patients with active pulmonary tuberculosis. Can J Public Health.

[B38] Espinal MA, Laszlo A, Simonsen L, Boulahbal F, Kim SJ, Reniero A, Hoffner S, Rieder HL, Binkin N, Dye C, Williams R, Raviglione MC, for the World Health Organization-International Union against Tuberculosis and Lung Disease Working Group on Anti-Tuberculosis Drug Resistance Surveillance (2001). Global trends in resistance to antituberculosis drugs. N Engl J Med.

[B39] Granich RM, Balandrano S, Santaella AJ, Binkin J, Castro KG, Marquez-Fiol A, Anzaldo G, Zarate M, Jaimes ML, Velazquez-Monroy O, Salazar L, Alvarez-Lucas C, Kuri P, Flisser A, Santos-Preciado J, Ruiz-Matus C, Tapia-Conyer R, Tappero JW (2000). Survey of drug resistance of Mycobacterium tuberculosis in 3 Mexican states, 1997. Arch Intern Med.

[B40] Espinal MA, Báez J, Soriano G, Garcia V, Laszlo A, Reingold AL, Sanchez S (1998). Drug-resistant tuberculosis in the Dominican Republic: results of a nationwide survey. Int J Tuberc Lung Dis.

[B41] Pitchenik AE, Russell BW, Cleary T, Pejovic I, Cole C, Snider DE (1982). The prevalence of tuberculosis and drug resistance among Haitians. N Engl J Med.

[B42] Brown RE, Miller B, Taylor WR, Palmer C, Bosco L, Nicola RM, Zelinger J, Simpson K (1995). Health-care expenditures for tuberculosis in the United States. Arch Intern Med.

[B43] Kopanoff DE, Snider DE, Caras GJ (1978). Isoniazid-related hepatitis: a U.S. Public Health Service cooperative surveillance study. Am Rev Respir Dis.

[B44] Ormerod LP, Horsfield N (1996). Frequency and type of reactions to antituberculosis drugs: observations in routine treatment. Tuber Lung Dis.

[B45] Nolan CM, Goldberg SV, Buskin SE (1999). Hepatotoxicity associated with isoniazid preventive therapy: a 7-year survey from a public health tuberculosis clinic. JAMA.

[B46] Menzies D, Dion MJ, Rabinovitch B, Mannix S, Brassard P, Schwartzman K (2004). Treatment completion and costs of a randomized trial of rifampin for 4 months versus isoniazid for 9 months. Am J Respir Crit Care Med.

[B47] Markowitz N, Hansen NI, Wilcosky TC, Hopewell PC, Glassroth J, Kvale PA, Mangura BT, Osmond D, Wallace JM, Rosen MJ, Reichman LB (1993). Tuberculin and anergy testing in HIV-seropositive and HIV-seronegative persons. Pulmonary Complications of HIV Infection Study Group. Ann Intern Med.

[B48] Al Zahrani K, Al Jahdali H, Menzies D (2000). Does size matter? Utility of size of tuberculin reactions for the diagnosis of mycobacterial disease. Am J Respir Crit Care Med.

[B49] Ferebee SH, Mount FW, Murray FJ, Livesay VT (1963). A controlled trial of isoniazid prophylaxis in mental institutions. Am Rev Respir Dis.

[B50] Horwitz O, Wilbek E, Erickson PA (1969). Epidemiological basis of tuberculosis eradication. Longitudinal studies on the risk of tuberculosis in the general population of a low-prevalence area. Bull World Health Organ.

[B51] Burman WJ, Stone BL, Reves RR, Wilson ML, Yang Z, el-Hajj H (1997). The incidence of false-positive cultures for Mycobacterium tuberculosis. Am J Respir Crit Care Med.

[B52] Long R, Maycher B, Scalcini M, Manfreda J (1991). The chest roentgenogram in pulmonary tuberculosis patients seropositive for human immunodeficiency virus type 1. Chest.

[B53] Rivest P, Ministère de la Santé et des Services sociaux du Quebec, Comité Québecois sur la Tuberculose, Bureau de surveillance épidémiologique, Direction de santé publique de Montréal-Centre Épidémiologie de la tuberculose au Québec de 2000 à 2003. Ministère de la Santé et des Services sociaux, Publications 2006.

[B54] Khan K, Muennig P, Behta M, Zivin JG (2002). Global drug-resistance patterns and the management of latent tuberculosis infection in immigrants to the United States. N Engl J Med.

[B55] Moore RD, Chaulk CP, Griffiths R, Cavalcante S, Chaisson RE (1996). Cost-effectiveness of directly observed versus self-administered therapy for tuberculosis. Am J Respir Crit Care Med.

[B56] Wurtz R, White WD (1999). The cost of tuberculosis: utilization and estimated charges for the diagnosis and treatment of tuberculosis in a public health system. Int J Tuberc Lung Dis.

[B57] Taylor Z, Marks SM, Rios Burrows NM, Weis SE, Stricof RL, Miller B (2000). Causes and costs of hospitalization of tuberculosis patients in the United States. Int J Tuberc Lung Dis.

